# Identifying the role of Phytomolecules in  the management of liver diseases by modulating NRF2 pathway: A Scoping Review Protocol

**DOI:** 10.12688/f1000research.150635.2

**Published:** 2024-07-08

**Authors:** Ajay Mili, Priyobrat Rajkhowa, Krishnadas Nandakumar, Richard Lobo

**Affiliations:** 1 Pharmacognosy, Manipal Academy of Higher Education, Manipal, Karnataka, 576104, India; 2 Health Policy, Prasanna School of Public Health, Manipal Academy of Higher Education, Manipal, Karnataka, 576104, India; 3 Pharmacology, Manipal College of Pharmaceutical Sciences, Manipal Academy of Higher Education, Manipal, Karnataka, 576104, India

**Keywords:** NRF2 pathway, Liver diseases, Phytomolecules, Oxidative stress

## Abstract

**Background:**

The Liver is a vital organ in the human body, which plays a crucial role in various physiological processes. Oxidative stress is a critical factor in the pathogenesis and progression of various liver diseases, contributing to cellular damage and dysfunction. The Liver is particularly vulnerable to the harmful effects of reactive oxygen species when the balance between their production and the body’s antioxidant defense mechanisms is disrupted. The nuclear factor erythroid 2-related factor 2 (NRF2) pathway has emerged as a promising therapeutic target for liver diseases due to its pivotal role in cellular defense against oxidative stress and inflammation. Plants have always been a source of drugs which has been used to treat various pharmacological disorders and most of its activity is due to its potential as an antioxidant. However, the specific mechanisms by which they interact with the NRF2 pathway and confer protection against liver diseases remain inadequately elucidated. Therefore, this scoping review aims to identify and analyze the existing literature pertaining to the relationship between Phytomolecules, which can modulate NRF2 and protect against liver diseases.

**Methods:**

The proposed scoping review will follow the steps given by “Arksey and O’Malley and Levac et al”. Electronic databases (PubMed/MEDLINE, Embase, etc.) will be searched for recent relevant studies. A predefined criterion for the inclusion and exclusion of studies will be independently adopted by two reviewers. The review will be presented as per the “Preferred Reporting Items for Systematic Reviews and Meta-analyses Extension for Scoping Review (PRISMA-ScR)” guidelines.

**Conclusion:**

The scoping review finding is expected to help understanding the role of Phytomolecules in preventing liver diseases by modulating the NRF2 pathway. Ultimately, this review will serve as a foundational step toward developing targeted interventions to improve liver health outcomes and reduce the global burden of liver diseases.

## Introduction

Liver diseases represent a significant global health challenge, including a variety diseases including viral hepatitis, alcoholic liver disease, non-alcoholic fatty liver disease (NAFLD), drug-induced liver injury (DILI), and liver cirrhosis. ^1^^,^^2^ The liver, a pivotal metabolic organ, plays an important role in physiological processes including detoxification, metabolic regulation, protein biosynthesis, bile secretion, and immunomodulation. Intracellular reactive oxygen species (ROS) are produced internally, for instance during mitochondrial oxidative phosphorylation, or can emerge through interactions with external agents such as xenobiotic substances. For maintain the ROS levels, Liver has various antioxidant enzymes, such as glutathione peroxidase, catalase, and superoxide dismutase (SOD). Any imbalance in the working of the antioxidant enzymes can lead to oxidative stress and it is one of the key factors in the progression and onset of liver diseases. ^3^^,^^4^

The Nuclear factor erythroid 2-related factor 2 (NRF2) pathway has emerged as a promising target for managing liver diseases. NRF2, a transcription factor, is integral to cellular defense mechanisms against oxidative stress and inflammation. Upon activation, NRF2 facilitates the expression of various genes involved in antioxidant and detoxification processes, thereby increasing the synthesis of antioxidant enzymes and proteins that mitigate the negative impacts of ROS and pro-inflammatory mediators. Growing evidence implicates NRF2 pathway dysregulation in liver disease pathogenesis and progression. Both experimental and clinical investigations have explored NRF2 modulation to mitigate liver damage, attenuate inflammation, and enhance hepatocyte viability. ^5^^,^^6^ Consequently, identifying compounds capable of activating the NRF2 pathway holds potential for advancing liver disease management strategies.

Phytomolecules, also known as phytochemicals or secondary metabolites, have recently been gaining a distinguished status as a potential source of drugs for treating various diseases. Phytomolecules have various pharmacological activities, such as antioxidant, ^7^^,^^8^ anti-inflammatory, ^7^ anticancer, ^9^^,^^10^ Cardioprotective, ^11^^,^^12^ Neuroprotective, ^13^^,^^14^ Hepatoprotective, ^15^^,^^16^ through their potential as an antioxidant and the potential to alter the signaling pathways.

It is essential to note that the precise role of Phytomolecules as modulators of the NRF2 pathway in managing liver diseases remains uncertain or ambiguous. Consequently, this scoping review is being undertaken to address this knowledge gap. By amalgamating existing literature on the role of Phytomolecules as modulators of the NRF2 pathway in managing liver diseases through the adoption of scoping review methodology, this study distinguishes itself and contributes novel evidence to the field.

## Protocol

### Methods

The protocol for the scoping review has been formulated by utilizing the frameworks proposed by “Arksey and O’Malley and Levac et al.”. ^17^ This review will adhere to the guidelines outlined in the Preferred Reporting Item for Scoping Reviews (PRISMA-ScR). For ensuring proper reporting, the “Preferred Reporting Items for Scoping Reviews (PRISMA-ScR)” will be adopted. ^18^

### Review question

The review question was framed through systematic brainstorming and iteratively refining ideas by the team. A literature search on Phytomolecules, NRF2 and Liver disease was done, leading to our research question.

1. What are the different types of Phytomolecules that contribute to the modulation of the NRF2 pathway for protection against Liver disease?

### Eligibility criteria

JBI manual for evidence synthesis 2020 format will be adopted for the development of research question PCC (Population, Concept, Context) ( Table 1). ^19^ The study’s primary goal is to methodically identify and map the existing literature on the role of Phytomolecules in managing liver diseases through the modulation of the NRF2 pathway.

**Table 1.** PCC framework.

Population (P)	The population of interest refers to the individuals or animals (rats or mice) being studied. In this case, the population would include individuals or animals with liver diseases. Liver diseases encompass a range of conditions, such as liver fibrosis, cirrhosis, hepatitis, non-alcoholic fatty liver disease (NAFLD), and others.
Concept (C)	The scoping review will explore the Phytomolecules and their role in modulating the NRF2 pathway. Also, management of liver diseases through the use of Phytomolecules.
Context (C)	The context of human and animal studies is chosen to establish the relevance of NRF2 in a controlled experimental setting.

### Search strategy

A literature search will be done in the electronic database, including Scopus, Web of science, CINAHL, PubMed, and EMBASE, to identify recent relevant literature published from 2015 to 2024. The keywords which will be used for searching are listed in Table 2. The search will focus exclusively on articles written in English. We’ll conduct a comprehensive exploration of potentially relevant articles by scrutinizing the reference lists of the included papers and any available grey literature. Rayyan, an online platform, will be utilized to manage the collected data. ^20^

**Table 2.** Search terms.

Population: Liver disease	Concept 1: NRF2 pathway	Concept 2: Natural compounds
Non-alcoholic fatty Liver Non-alcoholic steatohepatitis Alcoholic fatty liver disease Alagille syndrome Biliary Atresia Cirrhosis Hemochromatosis Hepatitis Primary Biliary Cholangitis Cirrhosis Primary Sclerosing Cholangitis Porphyria Wilson Disease Drug induced liver injury Chemical induced liver injury	NRF2 Nuclear factor erythroid 2-related factor 2 Nuclear factor erythroid-derived 2-like 2 NFE2L2	Natural compound * Natural product * Extract * Natural molecule * Plant based compound * Phytomolecule * Phytocompound Botanical compound * Phytochemical * Herbal compound * Phytonutrient * Secondary metabolite *

* Wildcard character of Boolean search to include alternative form of word.

Scoping reviews are broad by nature, so searching, screening, and selecting studies can uncover new terms, ideas, and even sources of information that weren’t initially considered. The search strategy can be modified throughout the review process to keep up with these findings.

### Study selection

All identified citations will be collected and uploaded into Rayyan, with duplicates being removed post-search. Two reviewers (AM & PR) will screen all retrieved titles and abstracts and assess the papers for eligibility based on predefined inclusion criteria. In cases where the reviewers are uncertain about a study's eligibility, a third reviewer (RL) or an independent opinion may be sought. The authors will reach a consensus on potentially relevant research through collective screening, and the full text of these studies will be retrieved for further review by agreement.

Included studies will undergo data charting and be reported by adhering to the PRISMA 2020 diagram. ^21^ All studies meeting the inclusion and exclusion criteria, regardless of quality, will be incorporated ( Table 3).

**Table 3.** Inclusion and exclusion criteria.

Inclusion criteria	Exclusion criteria
Studies investigating the role of nuclear factor erythroid 2-related factor 2 (NRF2) modulation in the management, prevention, or treatment of liver diseases.	Studies that do not specifically address the role of NRF2 in the management of liver diseases should be excluded.
Both experimental and observational studies, including randomized controlled trials, cohort studies, case-control studies, cross-sectional studies, case series and animal studies to be considered.	Any reviews, commentaries, Conference abstract presentations to be excluded.
Studies published in any language will be included. Non-English articles will be translated using online tools; if accurate translation is not possible, the study will be excluded, and this limitation will be duly noted in the limitations section.	

### Data charting

The process of data charting will be executed utilizing a predetermined format designed for this purpose. Two reviewers will independently chart data for each paper included in the review using Microsoft excel. In the case of any disagreement, the opinion of an additional or the third reviewer will be sought. This systematic procedure will assist in identifying gaps in the research area. The data extraction will include i) Study title, ii) Authors, iii) Publication year, iv) Study design, v) Study objectives, vi) Findings, vii) Study subjects, viii) Liver diseases studied, ix) Type of phytomolecules, x) Phytomolecule source, xi) NRF2 pathway modulation, xii) Outcome measures, xiii) Conclusion, and xiv) Recommendations.

The data extraction protocol will be iteratively refined as data is obtained from each included source of evidence. Any adjustments made will be meticulously documented within the scoping review. As a pragmatic measure, there will be no direct outreach to included study authors to solicit missing or supplementary data.

### Collating, summarizing, and reporting the results

The study findings will be synthesized using a combination of narrative (qualitative analysis) and tabular formats to present the generated evidence. Qualitative and quantitative evidence from the developed data charting table will help in mapping the accessible literature and reporting studies that depict the key findings pertaining to the roles of Phytomolecules as NRF2 modulator in the management of liver diseases. The process will involve identifying knowledge gaps or areas necessitating further research. Additionally, consider disseminating the findings through publication in a peer-reviewed journal or presentation at relevant conferences.

## Discussion

Throughout history, humans have utilized plants as a source of beneficial molecules, including Phytomolecules. Therefore, the present scoping review seeks to identify and document the Phytomolecules that can protect against liver disease by modulating the NRF2 pathway. The review’s findings will help provide a comprehensive overview of the Plant, its potential Phytomolecules, class of Phytomolecules, and the type of liver disease that can be treated or managed by Phytomolecules by NRF2 modulation. By providing early insights into a drug’s potential and guiding its development, they significantly increase the chances of success while ensuring ethical considerations and regulatory compliance, paving the way for developing safe and effective treatments.

The limitations of this review are that it excludes non-English databases in the literature search as it can cause practical implications, and neither will assess the quality of studies methodology.

### Dissemination

Findings will be disseminated in a scientific journal.

### Study status

The literature search is ongoing.

### Ethical considerations

As there are no human participants involved, there will be no requirement for ethical approval. Patients and/or the public were not involved in the design of this protocol.

## Authors contribution

Ajay Mili: Conceptualization, Methodology, Investigation, Data Curation, Writing- Original draft preparation, Writing-Reviewing and Editing. Priyobrat Rajkhowa: Methodology, Investigation, Data Curation, Writing-Reviewing and Editing. Krishnadas Nandakumar: Supervision, Data Curation, Writing-Reviewing and Editing. Richard Lobo: Supervision, Data Curation, Writing-Reviewing and Editing.

## Data Availability

No data are associated with this article. Open Science Framework: “PRISMA-ScR-Fillable-Checklist_10Sept2019”, https://doi.org/10.17605/OSF.IO/C3P8K.
