# Supplementary material for: Identifying the role of Phytomolecules in  the management of liver diseases by modulating NRF2 pathway: A Scoping Review Protocol
Source: F1000Res. 2024 Jul 8;13:522. Originally published 2024 May 22. [Version 2] doi: 10.12688/f1000research.150635.2 (PMC12015427; doi:10.12688/f1000research.150635.2)
Supplement: Peer review history

# Reviewer report: Reviewer response for version 2

**DOI:** 10.5256/f1000research.168761.r313388

**Published:** 2025-04-22

**Authors:** Anju Dhiman

**Affiliations:** 1 Department of Pharmaceutical Sciences, Maharshi Dayanand University Rohtak, Rohtak, Haryana, India

The article titled "Identifying the role of Phyto-molecules in the management of liver disease by modulating NRF2 pathway: A Scoping Review Protocol" outlines a comprehensive review protocol aimed at exploring the therapeutic potential of phyto-molecules in liver disease management. Specifically, it focuses on the role of the NRF2 pathway, which is known for its protective effects against oxidative stress and inflammation in liver diseases. The review protocol aims to systematically examine existing evidence on how various plant-derived compounds can modulate the NRF2 pathway and contribute to liver health. Given the increasing interest in natural products as alternative or adjunctive treatments for liver diseases, this article is highly relevant for advancing research in this field.

**Major Points:**

 Clarity and Structure of the Protocol:

 The protocol is well-organized, with clear sections outlining the objectives, methodology, and expected outcomes. The systematic approach to reviewing the literature on phyto-molecules and NRF2 is commendable, as it provides a structured framework for the review process.

 Importance of NRF2 in Liver Disease:

 The focus on NRF2 as a target for liver disease therapy is highly relevant and timely. Given the central role of NRF2 in cellular defense mechanisms, its modulation through phyto-molecules offers promising therapeutic potential, especially in liver diseases that involve oxidative stress and inflammation.

 Selection of Phyto-molecules:

 The inclusion of a wide variety of plant-derived compounds provides a comprehensive perspective on the topic. The article addresses both commonly studied and lesser-known phyto-molecules, which will help broaden the scope of the review and offer new insights into liver disease management.

 Methodology:

 The methodology proposed for this scoping review is sound, and the inclusion/exclusion criteria are well-defined. The systematic search strategy ensures that the review will be thorough, and the article gives sufficient detail on how relevant studies will be selected and analyzed.

**Minor Points:**

 Justification for Choosing Phyto-molecules:

 While the protocol highlights the importance of phyto-molecules, a brief section discussing the rationale for focusing on these compounds over synthetic drugs could provide additional context. Phyto-molecules' potential advantages, such as fewer side effects, might strengthen the relevance of this study.

 Future Implications and Applications:

 The article could benefit from a brief discussion of the potential clinical implications of the findings. For instance, how the results could influence future drug development, clinical practices, or patient outcomes in liver disease management would provide a clearer picture of the long-term impact of the research.

 Language and Terminology:

 The language used in the article is generally clear, but some technical terms (e.g., "NRF2 pathway modulation") might be explained in simpler terms for a broader audience, particularly for non-experts in the field of molecular biology.

 Conclusion: In conclusion, the article presents a promising scoping review protocol that addresses an important and relevant topic. The study's approach to examining phyto-molecules in the context of the NRF2 pathway for liver disease management is well thought out, and the methodology appears rigorous. I believe this review will contribute significantly to the current body of knowledge in the field.

Is the study design appropriate for the research question?

Yes

Is the rationale for, and objectives of, the study clearly described?

Yes

Are sufficient details of the methods provided to allow replication by others?

Yes

Are the datasets clearly presented in a useable and accessible format?

Yes

Reviewer Expertise:

Phytomedicines

I confirm that I have read this submission and believe that I have an appropriate level of expertise to confirm that it is of an acceptable scientific standard.

# Reviewer report: Reviewer response for version 2

**DOI:** 10.5256/f1000research.168761.r300409

**Published:** 2024-07-12

**Authors:** Tamer A. Addissouky

**Affiliations:** 1 Menoufia University, Shebeen El-Kom, Menofia Governorate, Egypt

This manuscript can be indexed.

Is the study design appropriate for the research question?

Yes

Is the rationale for, and objectives of, the study clearly described?

Yes

Are sufficient details of the methods provided to allow replication by others?

Yes

Are the datasets clearly presented in a useable and accessible format?

Yes

Reviewer Expertise:

biomedical

I confirm that I have read this submission and believe that I have an appropriate level of expertise to confirm that it is of an acceptable scientific standard.

# Reviewer report: Reviewer response for version 1

**DOI:** 10.5256/f1000research.165225.r288724

**Published:** 2024-06-14

**Authors:** Tamer A. Addissouky

**Affiliations:** 1 Menoufia University, Shebeen El-Kom, Menofia Governorate, Egypt

Positive points:

 - The research question is clearly defined using the PCC framework which provides context and scope for the review.

 - An extensive search strategy is described using relevant databases and search terms to capture all available literature. 

 - Clear inclusion/exclusion criteria are defined to filter relevant studies in a systematic manner.

 - A team-based approach is outlined for the different stages of title/abstract screening, full-text review, and data extraction to ensure objectivity.

 - Data will be charted using a standardized format and then summarised both narratively and quantitatively to synthesise evidence.

 - Findings will be disseminated through publication, adding value.

 Negative points:  

 - Quality assessment of studies is not mentioned which is important to evaluate strength of evidence.

 - No mention of handling any disagreement between reviewers during study selection.

 - Only English literature will be included which can introduce language bias.

 - No plans to search grey literature sources which may contain some relevant data.

 - No qualitative analysis is proposed which could provide contextual insights beyond numbers.

 Recommendations:

 - Develop and apply a quality assessment tool to appraise rigour of primary studies. 

 - Detail a clear process for resolving any disagreements between reviewers.

 - Consider translating the abstracts of relevant non-English studies to broaden scope.

 - Include searches of dissertations, conference abstracts and trials registries.

 - Use qualitative data analysis methods like thematic synthesis for narrative findings.

 - Engage stakeholders to obtain their perspectives and ensure findings are meaningful.

 - Pilot test all tools and processes on a sample of studies to address any issues early.

 In summary, this protocol outlines a rigorous systematic approach. Enhancing the quality assessment process, broadening the scope and applying qualitative synthesis would strengthen the review.

Is the study design appropriate for the research question?

Yes

Is the rationale for, and objectives of, the study clearly described?

Yes

Are sufficient details of the methods provided to allow replication by others?

Yes

Are the datasets clearly presented in a useable and accessible format?

Yes

Reviewer Expertise:

biomedical

I confirm that I have read this submission and believe that I have an appropriate level of expertise to confirm that it is of an acceptable scientific standard, however I have significant reservations, as outlined above.

# Response

**Published:** 2024-07-02

**Authors:** Richard Lobo

**Affiliations:** Manipal University, India

Dear Reviewer,

 Thank you for your detailed and constructive feedback on our scoping review protocol. We appreciate your insights and suggestions, which will undoubtedly strengthen the rigor and comprehensiveness of our study. Below are our responses and actions based on your specific points:

**Acknowledgment:** We acknowledge your positive feedback on several aspects of our protocol:

- The research question is clearly defined using the PCC framework, providing a robust context and scope.
- An extensive search strategy is outlined, utilizing relevant databases and search terms to comprehensively capture available literature.
- Clear inclusion/exclusion criteria are defined to systematically filter relevant studies.
- A team-based approach is planned for screening, review stages, and data extraction to ensure objectivity.
- Data will be charted using a standardized format and synthesized both narratively and quantitatively.
- Findings will be disseminated through publication, adding value to the existing literature.

**Responses to Specific Questions:**

1. **Quality Assessment of Studies:** We acknowledge your concern regarding the quality assessment of individual studies in our scoping review. Please note that our review is focused on mapping the breadth of literature rather than conducting a formal quality assessment. The methodology follows the Arksey, H., & O’Malley, L. (2005) framework, which does not include the adoption of a quality assessment for studies. We aim to adhere to this established methodology. Furthermore, this scoping review serves as a preliminary step toward a systematic review. Upon completion of the scoping review, we plan to conduct a systematic review where we will incorporate a quality assessment tool to evaluate the included studies.
2. **Handling Disagreements Between Reviewers:** We acknowledge your concern regarding the handling of disagreements between reviewers. This aspect is addressed in the study selection section of the methods section. We have now comprehensively detailed the process for handling disagreements between reviewers.
3. **Language Bias:** We acknowledge your comment regarding the inclusion of studies published in various languages. We will attempt to include studies published in any language in the scoping review. However, the translation of these studies will rely on online translation tools. If we are unable to accurately translate certain articles, they will be excluded from the review. This limitation will be duly acknowledged in the limitations section of the manuscript. We have mentioned our approach to including any language of studies in the scoping review in the manuscript in Table 3.
4. **Grey Literature:** We acknowledge the importance of including grey literature sources to capture additional relevant data. We have mentioned our approach to including grey literature in the manuscript in the search strategy section of the methods section.
5. **Qualitative Analysis:** We appreciate your emphasis on the importance of qualitative analysis. While we initially proposed only narrative analysis, we recognize the value of incorporating additional qualitative data analysis methods, such as thematic synthesis, to provide deeper contextual insights. We have addressed this in the “Collating, summarizing, and reporting the results” section of the manuscript.

**Response to recommendations:**

- **Develop and apply a quality assessment tool to appraise the rigor of primary studies:** We wish to clarify that our scoping review does not include a formal quality assessment of individual studies, as per the methodology outlined by Arksey and O’Malley (2005), which does not prescribe such an assessment. Implementing changes to this methodology at this stage poses challenges.
- **Detail a clear process for resolving any disagreements between reviewers:** We have embraced this recommendation and detailed a clear process for resolving disagreements between reviewers in the manuscript.
- **Consider translating abstracts of relevant non-English studies to broaden scope:** Regarding language bias, we have accepted the recommendation to consider translating abstracts of relevant non-English studies using online tools. If translation accuracy cannot be ensured, studies will be excluded as outlined in the manuscript.
- **Include searches of dissertations, conference abstracts, and trials registries:** We have accepted to include searches of grey literature sources such as dissertations, conference abstracts, and trial registries, as also specified in the manuscript.
- **Consider qualitative data analysis methods like thematic synthesis:** We have embraced the suggestion to incorporate qualitative data analysis methods like thematic synthesis, which has been highlighted in the manuscript.
- **Engage stakeholders to ensure findings are meaningful:** Upon completion of our study, we plan to consult various stakeholders to ensure that our findings are meaningful and impactful.
- **Pilot test all tools and processes on a sample of studies to address any issues early:** Recognizing its importance, we have conducted pilot testing on our tools, including the data extraction chart, to address any potential issues early in the process.

We are committed to enhancing the transparency, rigor, and inclusivity of our scoping review methodology based on your valuable feedback. Please do not hesitate to reach out if you have any further questions or suggestions.
